# Associations of Atopic Dermatitis in Late Adolescence with Occupation, Mental Health, Income from Work, and Marital Status: A National Longitudinal Study

**DOI:** 10.2340/actadv.v105.42127

**Published:** 2025-01-14

**Authors:** Grigorios THEODOSIOU, Therese STERNER, Ayako HIYOSHI, Michael CARLBERG, Åke SVENSSON, Scott MONTGOMERY, Laura VON KOBYLETZKI

**Affiliations:** 1 Department of Dermatology-Venereology, Skåne University Hospital (SUS), Malmö, Sweden; 2 Department of Clinical Sciences Malmö, Faculty of Medicine, Lund University, Malmö, Sweden; 3 Department of Care Science, Faculty of Health and Society Malmö University, Malmö Sweden; 4 Clinical Epidemiology and Biostatistics School of Medical Sciences, Faculty of Medicine and Health Örebro University, Örebro, Sweden; 5 Department of Epidemiology and Public Health, University College London, London, UK; 6 Clinical Epidemiology Division, Department of Medicine, Karolinska Institutet, Stockholm, Sweden; 7 School of Medical Sciences, Örebro University, Örebro, Sweden; 8 Department of Occupational and Environmental Dermatology, Lund University, Malmö, Sweden

**Keywords:** atopic dermatitis, epidemiology, quality of life

## Abstract

The main aim of this study was to evaluate longitudinal associations between atopic dermatitis (AD) in late adolescence and occupational socioeconomic group, labour market participation, income from work, and marital status later in adult life. Using Swedish population-based registry data a total of 205,394 men were included, born between 1952 and 1956 in Sweden and who underwent military conscription examination including assessment of atopic dermatitis (AD) and AD severity. The associations between AD and AD severity in late adolescence and labour market participation, income from work, marital status, and medication for anxiety and depression later in adult life were examined. In this study, men with AD in adolescence, especially those with severe AD, more often had a prescription for antidepressants or anxiolytics at the age of 50–57 years (unadjusted HR 1.55, 95% CI 1.32–1.81). Interestingly, despite increased risk of poorer mental health, AD was not found to be associated with a disadvantage in terms of occupational socioeconomic group, income from work, and unemployment benefits. Individuals with mild AD showed a lower risk of holding routine and lower technical jobs compared with men without AD. Persons with AD in late adolescence seem not to differ regarding registered partnerships and marital status compared with those without AD.

## SIGNIFICANCE

Using registry data, we included a total of 205,394 men born between 1952 and 1956 in Sweden who underwent military conscription examination including assessment of atopic dermatitis (AD) and AD severity. We examined the association between AD and AD severity in late adolescence and labour-market participation, income from work, marital status, and medication for anxiety and depression later in adult life. In this study, men with AD in adolescence, especially those with severe AD, more often had a prescription for antidepressants or anxiolytics at the age of 50–57 years. Despite this, AD was not found to be associated with a disadvantage in terms of occupational socioeconomic group, income from work, and unemployment benefits.

A topic dermatitis (AD) is a chronic and relapsing inflammatory skin disease with a 1-year prevalence of about 20% in children and adolescents, and 5% in adults (1, 2). Up to 50% of children, especially with severe disease, have exacerbations of AD during adulthood (3–6).

The main symptom of AD is itch with possible related sleeping problems, tiredness, and decreased concentration (7, 8). Further, visible flares may adversely influence aspects of self-confidence and social interaction (7–9). Due to the often debilitating nature of the condition, AD is related to the highest burden of disease related to skin conditions (10). AD implies significant societal economic burden, including direct healthcare costs as well as indirect costs for patients with AD (11–13).

AD is related to several comorbid conditions; about 30% of children with AD later develop asthma and allergic rhinitis (14). Recently, several non-atopic conditions such as the development of attention deficit hyperactivity disorder, a diagnosis of anxiety, and a diagnosis of depression were reported to be associated with AD (15, 16). It is plausible that AD itself, with physical disfigurement and possible problems with social interactions as well as itch and possible related sleep disturbance, might negatively influence educational and work performance. Comorbid mental health conditions might add to concentration difficulties, days off school, and work, due to symptoms and treatments.

A German cross-sectional study reported that severe AD was associated with more severe depression and a nationwide Danish cohort study reported a positive association between moderate-to-severe AD and use of antidepressants (17, 18). A previous study using the same population found that AD in adolescence was associated with a greater risk of treatment for depression in middle age but did not examine the difference in risk by AD severity (19).

Despite rapidly increasing research in AD, observational studies examining the clinical burden of AD have been scarce. It has been reported that there is no association between AD and educational attainment in a longitudinal cohort study of men who underwent military conscription examination between the years 1969 and 1976 (20). In another cohort study, including over 10,000 children with AD identified and followed using a Danish database, it was found that patients diagnosed with AD in childhood had a lower likelihood of completing further academic education later in life, particularly those with severe AD (21). This is in line with our recent cross-sectional study, where we found that adults with severe AD were less likely to have a university degree or higher qualification (22).

The aim of this study was to examine the relationship between AD in adolescence, labour market participation, unemployment benefits, income from work, and marital status among males in Sweden. Although our previous study found that AD in adolescence was associated with a greater risk of treatment for depression in middle age (16), we re-examined this association because the previous study had not examined the difference in risk by the severity of AD.

## MATERIALS AND METHODS

### Study population and design

**Fig. 1:**
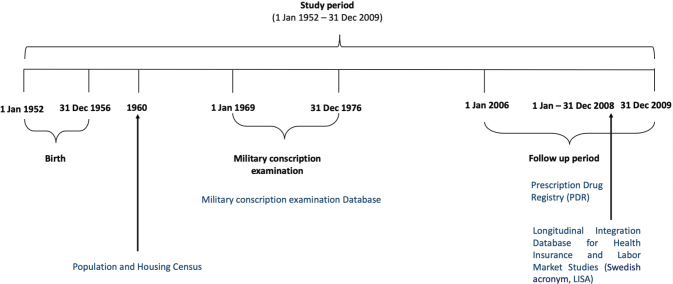
Schematic of study design.

This is a cohort study with prospectively recorded data on men who were born in 1952–1956 and attended military conscription examinations in Sweden at age 17–21 years during the years 1969–1976. For this cohort, conscription was compulsory except for individuals with severe disability or who were incarcerated. Exclusion criteria for the study were men with missing data in variables used in this study, mainly due to unsuitability for military service, emigration or death before the start of follow-up, and those who did not have the conscription examination at age 17–21 years. The conscription examination, which included medical history, a medical examination, various physical and psychological tests, as well as a cognitive function test, has been described in detail in a previous article by Smirnnova et al. (20). Data from the military conscription examinations were linked to national registers with follow-up until 31 December 2009. The study design is presented in **Fig. 1**.

### Exposure

The exposure variable was AD and the severity of AD. Information on AD was collected by a physician during a general physical examination and a medical history assessment during the conscription assessment. The Swedish International Classification of Diseases 8th Revision (ICD-8) code 691 was used to identify AD.

Severity of atopic dermatitis was defined using the seventh digit of the disease code recorded in the conscription data to indicate the severity of disease (from a military-medical point of view) by 8 levels. Codes 5–8 (“fairly significant impairment” to “no impairments, meaningless defect”) were defined as “mild” (*n* = 1,277) and codes 0–4 (“highly significant impairment” to “considerable impairment”) were defined as “severe” (*n* = 181).

### Outcome variables

The information on occupational socioeconomic group in 2008 was assessed using an occupational socioeconomic classification approximating ESeC with 7 groups: (*i*) large employers/higher managers/professionals; (*ii*) lower managers/professionals, higher supervisory/technicians; (*iii*) intermediate occupations; (*iv*) lower supervisors and technicians; (*v*) lower sales and service; (*vi*) lower technical, and (*vii*) routine.

Labour market participation status was grouped in 3 categories, as recorded in the LISA register: (*i*) Working in November 2008; (*ii*) Not working in November 2008, and (*iii*) Not worked at all during 2008. Any unemployment benefits during the whole year (yes/no) were also assessed. Income was assessed by gross salary in 2008. Income from work was grouped in 4 groups: (*i*) ≤ SEK244,500 (< €25,451) (*ii*) SEK244,600–322,300 (€25,462–33,550) (*iii*) 322,400–420,000 (€33,560–43,720), (*iv*). > SEK420,000 (> €43,720).

For marital status, data from the LISA register were used for assessment of the partnership status at the end of 2008. The data were categorized into 3 groups: (*i*) Never married/registered partner; (*ii*) Married/registered partner, widowed/partner died; and (*iii*) Divorced.

Using the Prescribed Drug Registry (PDR), which includes data on all pharmacy-dispensed medications, and pharmacy dispensation dates, from 1 January 2006 until 31 December 2009, the first date on which the sum of dispensed anxiolytics or antidepressants became equivalent to their use for more than or equal to 180 days within 365 days was taken as the incidence date, indicating continuous treatment for anxiety or depression over an extended period.

### Statistical analysis

Cross-tabulation and median and mean were used to describe the study population and distribution of data as appropriate. The exposure was AD (any AD; mild, severe); the main outcomes were occupational group, employment status, income, unemployment benefits, and marital status in 2008. The relationship between the AD and outcomes was examined by logistic regression for dichotomous outcome (unemployment benefits), and multinomial regression for outcomes with more than 2 categories (occupational group, gross salary, marital status, employment status in November 2008). Coefficients, odds ratios (OR), and relative risk ratios (RRR) were estimated from these regression models, respectively, with 95% confidence intervals (95% CI). To examine the association between AD and depression and anxiety, we used Cox regression, estimating hazard ratios (HR). The proportional hazards assumption was examined using Schoenfeld residuals, and no evidence of violation was found. We stratified the analyses between AD and the outcome variables by comorbidity status to examine whether the association may differ by having any comorbid disease.

A p-value lower than 0.05 or 95% confidence intervals not including 1.00 indicated statistical significance. The analyses were conducted using Stata version 15.1 (StataCorp LLC, College Station, TX, USA).

Data, ethics, covariates are presented in Appendix S1.

## RESULTS

### Description of study population

The study population consisted of 205,394 men who underwent conscription assessment, of whom 1,458 (0.71%; 95% CI 0.67–0.75%) had a diagnosis of AD at conscription assessment. The study population is described in **Table I**.

**Table I:** Population description by atopic dermatitis diagnosis for 203 396 male adolescents assessed for military conscription between 1969 and 1976 in Sweden

Item	No AD		AD		Total	

	*n*	%	*n*	%	*n*	%
All	203,396		1,458		205,394	
Year of birth						
1952	39,717	19.5	199	13.6	39,916	19.4
1953	39,284	19.3	215	14.7	39,499	19.2
1954	40,557	19.9	240	16.5	40,797	19.9
1955	41,635	20.4	356	24.4	41,991	20.4
1956	42,743	21.0	448	30.7	43,191	21.0
Stress resilience						
Low (1–3)	39,491	19.4	352	24.1	39,843	19.4
Intermediate (4–6)	114,033	55.9	783	53.7	114,816	55.9
High (7–9)	50,412	24.7	323	22.2	50,735	24.7
Cognitive function score						
Mean (SD)	5.3	(1.9)	5.4	(2.0)	5.3	(1.9)
Any diagnosis of mental illness						
No mental illness	182,155	89.3	1,285	88.1	183,440	89.3
Mental illness	21,781	10.7	173	11.9	21,954	10.7
Asthma						
No asthma	200,175	98.2	1,213	83.2	201,388	98.0
Asthma	3,761	1.8	245	16.8	4,006	2.0
Head of household’s occupation						
Manual workers	84,233	41.3	554	38.0	84,787	41.3
Agricultural workers	7,957	3.9	35	2.4	7,992	3.9
Farm owners/managers	20,872	10.2	116	8.0	20988	10.2
Office workers	57,210	28.1	511	35.0	57,721	28.1
Business owners/managers	21,636	10.6	180	12.3	21,816	10.6
Others/unknown	12,028	5.9	62	4.3	12,090	5.9
Occupational socioeconomic classification*						
Large employers, higher managers/professionals	32,977	16.2	290	19.9	33,267	16.2
Lower managers/professionals, higher supervisory/technicians	46,149	22.6	351	24.1	46,500	22.6
Intermediate occupations	19,499	9.6	138	9.5	19,637	9.6
Lower supervisors and technicians	2,099	1.0	15	1.0	2,114	1.0
Lower sales and service	16,654	8.2	127	8.7	16,781	8.2
Lower technical	45,232	22.2	272	18.7	45,504	22.2
Routine	41,326	20.3	265	18.2	41,591	20.2
Gross salary (quartiles) (SEK)*						
≤ 244,500	50,445	24.7	353	24.2	50,798	24.7
244,600–322,300	51,136	25.1	342	23.5	51,478	25.1
322,400–420,000	51,213	25.1	360	24.7	51,573	25.1
> 420,000	51,142	25.1	403	27.6	51,545	25.1
Unemployment benefits*						
No benefits	195,208	95.7	1,400	96.0	196,608	95.7
Benefits	8,728	4.3	58	4.0	8,786	4.3
Employment status in November 2008*						
Working	189,616	93.0	1,369	93.9	190,985	93.0
Not working in November, has worked during the year	5,517	2.7	33	2.3	5,550	2.7
Not working in November, has not worked during the year	8,803	4.3	56	3.8	8,859	4.3
Marital status*						
Married**	118,886	58.3	844	57.9	119,730	58.3
Never married	51,089	25.1	387	26.5	51,476	25.1
Divorced	33,961	16.7	227	15.6	34,188	16.6

* LISA 2008 was used for information regarding occupational socioeconomic classification in 2008, gross salary, unemployment benefits, employment status in November 2008, and marital status.

** Including widowers.

Of the men diagnosed with AD at the conscription assessment, most had mild AD (*n* = 1,277, 87.6%; 95% CI 85.8–89.2%), and most had 1 or more comorbid conditions (*n* = 1,043, 71%; 95% CI 69.1–73.8%). Men with AD had a much higher prevalence of asthma (*n* = 245, 16.8%; 95% CI 14.9–18.8), tended to have lower stress resilience (*n* = 352, 24.1%; 95% CI 22.0–26.4%), and a similar prevalence of mental illness (*n* = 173, 11.9%; 95% CI 10.2–13.6) compared with those without AD. Further, men with AD were more often from families where the head of the household held a position with office work, compared with men without AD (35%; 95% CI 32.6–37.6%, *n* = 511 vs 28.1%; 27.9–28.2%, *n* = 52,210; respectively) (Table I).

### Atopic dermatitis and mental conditions

In total, 14,868 had an outcome event (antidepressant/anxiolytic medication); the median follow-up was 162 days (IQR 48–653 days). The results from unadjusted Cox regression showed that men with AD had more often prescribed antidepressant/anxiolytic medication at the age of 50–57 years (HR 1.55, 95% CI 1.32–1.81) compared with men without AD **(****Table II** **)**. Compared with individuals without AD, the risk of prescribed antidepressants and/or anxiolytics showed a dose–response association: HR was 1.42 for men with mild AD (95% CI 1.20–1.69) and 2.46 (95% CI 1.72–3.53) for men with severe AD.

**Table II:** Association between AD and prescription for antidepressants/anxiolytics between years 2006–2009

Factor	Medication/total no.	Unadjusted HR (95% CI)	Adjusted HR (95% CI)*
No AD	14,709/203,936	Reference	Reference
AD	159/1,458	1.55 (1.32–1.81)	1.49 (1.28–1.74)
–Mild AD	129/1,277	1.42 (1.20–1.69)	1.37 (1.16–1.63)
–Severe AD	30/181	2.46 (1.72–3.53)	2.36 (1.65–3.38)
AD without comorbidity			
No AD	14,709/203,936	Reference	Reference
AD	39/415	1.32 (0.96–1.81)	1.42 (1.03–1.94)
–Mild AD	33/365	1.26 (0.90–1.78)	1.35 (0.96–1.91)
–Severe AD	6/50	1.74 (0.78–3.88)	1.88 (0.85–4.19)
AD with comorbidity			
No AD	14,709/203,936	Reference	Reference
AD	120/1,043	1.64 (1.37–1.96)	1.52 (1.27–1.82)
–Mild AD	96/912	1.49 (1.22–1.82)	1.38 (1.13–1.69)
–Severe AD	24/131	2.75 (1.84–4.10)	2.52 (1.69–3.76)

Analysis used Cox regression to produce hazard ratios (HR).

* Adjusted for year of birth, cognitive function, any diagnosis of mental illness, and head of household’s occupation, and stress resilience.

### Atopic dermatitis and occupational socioeconomic classification

The results for the association between AD and occupational socioeconomic classification using multinomial logistic regression showed that, in 2008, men with AD had a lower risk of holding lower technical (unadjusted RRR 0.68, 95% CI 0.58–0.81) and routine occupations (unadjusted RRR 0.73, 95% CI 0.62–0.86) compared with men without AD in adolescence **(Table III)**. After adjusting for year of birth, cognitive function, any diagnosis of mental illness, and head of household’s occupation as well as stress resilience, the association remained similar.

**Table III:** Association between any atopic dermatitis at conscription examination and occupation, salary, unemployment benefits, employment, and marital status

Outcome variable (*n* = 205,394)	Unadjusted RRR (95% CI)	Adjusted RRR (95% CI)*
Occupational socioeconomic classification		
Large employers, higher managers/professionals	Reference	Reference
Lower managers/professionals, higher supervisory/technicians	0.86 (0.74–1.01)	0.88 (0.75–1.04)
Intermediate occupations	0.80 (0.66–0.99)	0.82 (0.67–1.01)
Lower supervisors and technicians	0.81 (0.48–1.37)	0.81 (0.48–1.36)
Lower sales and service	0.87 (0.70–1.07)	0.87 (0.70–1.08)
Lower technical	0.68 (0.58–0.81)	0.73 (0.60–0.87)
Routine	0.73 (0.62–0.86)	0.74 (0.62–0.89)
Gross salary (quartiles) (SEK)		
≤ 244,500	Reference	Reference
244,600–322,300	0.96 (0.82–1.11)	0.97 (0.83–1.12)
322,400–420,000	1.00 (0.87–1.16)	1.03 (0.89–1.19)
> 420,000	1.13 (0.98–1.30)	1.15 (0.99–1.34)
Unemployment benefits**		
No benefits	Reference	Reference
Benefits	0.93 (0.71–1.21)	0.93 (0.71–1.21)
Employment status		
Working	Reference	Reference
Not working in November, has worked during the year	0.83 (0.59–1.17)	0.86 (0.61–1.22)
Not working in November, has not worked during the year	0.88 (0.67–1.15)	0.87 (0.67–1.14)
Marital status		
Married	Reference	Reference
Never married	1.07 (0.95–1.20)	1.02 (0.90–1.16)
Divorced	0.94 (0.81–1.09)	0.93 (0.80–1.08)

Analysis used multinomial regression to produce relative risk ratios (RRR).

* Adjusted for stress resilience, year of birth, cognitive function, any diagnosis of mental illness, and head of household’s occupation.

** Logistic regression (OR, 95% confidence interval (CI)).

### Atopic dermatitis and labour market participation

No association between AD and labour market participation in November 2008 was found in unadjusted (RRR 0.88, 95% CI 0.67–1.15) or adjusted analyses (aRRR 0.87 95% CI 0.67–1.14) (Table III). Analysis by AD severity showed an unadjusted RRR for not worked during the year of 0.79 for men with mild AD (95% CI 0.58–1.07) and 1.55 for individuals with severe disease (95% CI 0.86–2.78). Adjusted analyses for year of birth, cognitive function, any diagnosis of mental illness, and head of household’s occupation as well as further adjustment including stress resilience did not alter these estimates notably, which remained not statistically significant as shown in **Table IV**.

**Table IV:** Association between atopic dermatitis, occupation, salary, unemployment benefits, and marital status by severity of atopic dermatitis (AD)

Outcome variable	Mild AD Unadjusted RRR (95% CI)	Severe AD Unadjusted RRR (95% CI)	Mild AD Adjusted RRR (95% CI)*	Severe AD Adjusted RRR (95% CI)*
Occupational socioeconomic classification				
Large employers, higher managers/professionals	Reference	Reference	Reference	Reference
Lower managers/professionals, higher supervisory/technicians	0.84 (0.71–0.99)	1.14 (0.70–1.84)	0.86 (0.73–1.02)	1.10 (0.68–1.80)
Intermediate occupations	0.78 (0.63–0.97)	1.00 (0.54–1.86)	0.81 (0.65–1.00)	0.96 (0.51–1.80)
Lower supervisors and technicians	0.84 (0.49–1.43)	0.58 (0.08–4.28)	0.84 (0.49–1.44)	0.55 (0.07–4.03)
Lower sales and service	0.79 (0.63–0.99)	1.61 (0.92–2.83)	0.80 (0.63–1.01)	1.45 (0.80–2.63)
Lower technical	0.66 (0.55–0.78)	0.95 (0.57–1.56)	0.71 (0.58–0.86)	0.91 (0.53–1.57)
Routine	0.69 (0.58–0.83)	1.09 (0.67–1.80)	0.71 (0.59–0.87)	0.99 (0.58–1.71)
Gross salary (quartiles) (SEK)				
≤ 244,500	Reference	Reference	Reference	Reference
244,600–322,300	0.92 (0.78–1.08)	1.20 (0.81–1.76)	0.93 (0.79–1.09)	1.19 (0.81–1.76)
322,400–420,000	1.04 (0.89–1.22)	0.75 (0.49–1.16)	1.04 (0.89–1.22)	0.76 (0.49–1.18)
> 420,000	1.17 (1.00–1.36)	0.86 (0.57–1.31)	1.12 (0.95–1.32)	0.90 (0.58–1.40)
Unemployment benefits**				
No benefits	Reference	Reference	Reference	Reference
Benefits	0.95 (0.72–1.25)	0.77 (0.34–1.73)	0.96 (0.73–1.27)	0.72 (0.32–1.64)
Employment status				
Working	Reference	Reference	Reference	Reference
Not working in November, has worked during the year	0.89 (0.62–1.27)	0.41 (0.10–1.66)	0.93 (0.65–1.34)	0.39 (0.10–1.57)
Not working in November, has not worked during the year	0.79 (0.58–1.07)	1.55 (0.86–2.78)	0.79 (0.58–1.07)	1.43 (0.79–2.58)
Marital status				
Married	Reference	Reference	Reference	Reference
Never married	1.07 (0.94–1.21)	1.06 (0.75–1.50)	1.03 (0.90–1.17)	0.99 (0.70–1.41)
Divorced	0.93 (0.79–1.08)	1.05 (0.71–1.57)	0.92 (0.78–1.07)	1.01 (0.68–1.52)

Analysis used multinomial regression to produce relative risk ratios (RRR).

* Adjusted for year of birth, stress resilience, cognitive function, any diagnosis of mental illness, and head of household’s occupation.

** Logistic regression (OR, 95% confidence interval (CI)).

### Atopic dermatitis and unemployment benefits

Table III shows that AD was not statistically significantly associated with higher risk of receiving unemployment benefits in crude analysis (OR 0.93, 95% CI 0.71–1.21). Adjustment for year of birth, cognitive function, any diagnosis of mental illness, and SEI of the head of household as well as additional adjustment including stress resilience did not alter the estimate notably (RRR 0.95, 95% CI 0.73–1.24 and RRR 0.93, 95% CI 0.71–1.21, respectively).

### Atopic dermatitis and gross salary

Compared with a lower income, the unadjusted multinomial regression model showed no statistically significant increased relative risk with an income of SEK420,000 or higher for persons with AD in adolescence (RRR 1.13, 95% CI 0.98–1.30) compared with individuals without AD (Table III). Adjustment for year of birth, cognitive function, any diagnosis of mental illness, and SEI of the head of household as well as additional adjustment including stress resilience did not make noticeable changes in the estimates.

### Atopic dermatitis and marital status

The unadjusted analysis found that AD was not statistically significantly associated with marital status, as shown in Table III. The unadjusted multinomial logistic regression produced a relative risk ratio of 1.07 (95% CI 0.95–1.20) for “never been married” and 0.94 (95% CI 0.81–1.09) for “been divorced” respectively for men with AD – with married as reference category – compared with those without AD. After adjustment for year of birth, cognitive function, any diagnosis of mental illness, and head of household’s occupation, the estimates did not alter notably and remained statistically not significant.

The complete results section including analyses by comorbidities is presented in Appendix S1.

## DISCUSSION

### Statement of principal findings

The present study did not show that AD in late adolescence was associated with any labour market disadvantage even though AD was associated with a higher risk of having a prescription for anxiolytics or antidepressants at ages 50 to 57 years. AD was not associated with marital status.

Using this large general population-based cohort study of Swedish males born in the 1950s we found no association between AD and higher risk of lower occupational position or lower income later on. Interestingly, males with mild AD later showed a slightly higher income and lower risk of holding routine and lower technical occupations, probably because of socioeconomic confounding (23, 24). Further, no association was found between AD and employment status, unemployment benefits, and marital status in our cohort.

Low stress resilience in adolescence has been linked to a number of adverse outcomes later in life such as depression and anxiety in middle age (25). We considered stress resilience as a possible mediator on the pathway and adjusted for it in an additional step. The models were all adjusted for year of birth, cognitive function, any diagnosis of mental illness, and head of household’s occupation. Additionally, we adjusted for stress resilience. No difference regarding the estimates was found between the 2 models.

### Comparison with previous work

To the best of our knowledge there are no other longitudinal studies that have investigated the association of AD with occupation in Sweden. A systematic review performed in 2018 identified 12 studies on the relationship between AD and work life. With regard to sick leave, several studies almost unanimously reported increased frequency in AD patients. This review suggested that AD affects job choice, was associated with change or loss of job, sick leave, and even disability pensions for patients with severe AD (26).

The results of this study are consistent with previous studies that found an association between AD anxiety and depression in both childhood and adulthood (14, 15). Our finding are also in line with another study which examined the same population and found that hay fever and atopic dermatitis in adolescence were associated with an elevated risk of being prescribed antidepressants 3 decades later (16).

As previously reported, the study population with AD, especially with severe disease, was found to have an increased risk of antidepressant and anxiolytic medication in middle age (16). This is in line with the findings of a German cross-sectional study that reported severe AD was associated with more severe depression and a nationwide Danish cohort study which reported a positive association between moderate-to-severe AD and use of antidepressants (17, 18). The difference in risk by severity of AD is probably driven by the symptom burden, which causes intense itch and disturbed sleep, which in turn may affect the mental health of the patient (27, 28). We speculate that it is also possible that inflammation might hypothetically affect mental health in patients with severe AD (25).

A cross-sectional Swedish study conducted by our group in 2019 found that people with AD, especially with severe AD, were more often unemployed, more often on sick leave, more often never married, and more often felt lonely than adults without AD (22). The findings could not be confirmed in the present longitudinal study that did not find a statistically significant association between AD, unemployment, and marital status. The aforementioned cross-sectional study found that individuals with severe AD more often had a lower income compared with those without AD, which was not confirmed by the present study (22). The different study design and a cohort effect, as well as the assessment of AD in different phases of life of the 2 populations may explain the contradictory results of these studies.

### Appraisal of methods

To the best of our knowledge, this is one of the largest and most comprehensive prospective studies on the burden of AD and labour market participation and marriage in Sweden. By including disease severity, pre-existing health conditions present in late adolescence, and socioeconomic variables over time, the findings represent a thorough evaluation of the burden of AD in Swedish males in a real-world setting.

By using Swedish register data, we were able to identify key factors in terms of sub-groups within the studied population. Our study uses combined data from the military conscription assessment and national registries, which is essential for the longitudinal follow-up of patients with AD. This enables an assessment of the association of AD with occupation status, income, marital status, and mental health.

We also acknowledge that this study presents some potential limitations. The findings of our study are based only on men born in the 1950s. Therefore, our results may not be generalizable to women. The prevalence of AD in this study is low (0.7%), while most studies report a prevalence between 3% and 14%. A study conducted in 1948 on the Faroe Islands reported a prevalence of AD of 1.54% (29). It may also be argued that prevalence of AD in the Nordic countries in the late 1960s was significantly lower compared with prevalence of the disease in the 2020s (22). It is also plausible to assume that those with mild disease or those with AD which had resolved by adolescence may have been missed. The conscription examination follows a standardized protocol to evaluate whether a potential conscript is suitable for military service, so mild cases of AD may not be considered relevant to record. Furthermore, because the management of AD has advanced over the past decades, associations may differ in a population born more recently.

The possible existence of AD in the population not identified as having AD would arguably have biased the results towards the null hypothesis, i.e., that there is no difference between the groups, suggesting that the true difference between the groups may be higher. Another possibility, though, is that associations are of higher magnitude due to a greater likelihood of including men with more severe and persistent disease.

Assessment of psychiatric conditions was through medication data as a proxy and this measure has been validated previously (30). However, the use of PDR enabled us to identify only the patients who were being treated with drugs, while it is likely that there are many more patients living with anxiety or depression who have never received a diagnosis or treatment for these conditions. It is plausible to assume that this may have introduced a degree of misclassification bias in our results.

Residual confounding is always a possibility in observational studies. There are likely to be unmeasured potential confounding factors such as timely access to medical care, family support, and access to social support, which might have influenced the estimates.

### Mechanisms

This study showed an association of mild AD and lower risk of holding routine and lower technical jobs. Moreover, males diagnosed with atopic dermatitis at the time of conscription in our study later showed a slightly higher income, especially those with mild AD. Potentially, AD at the time of conscription was related to a socioeconomic advantage, which levelled out the possible influence of decreased stress resilience and comorbid depression. Another possible explanation might be that individuals with AD may choose jobs with less skin strain because of their skin disease and therefore tend to choose white-collar jobs instead of blue-collar jobs. This might be a possible explanation for the lack of economic disadvantage.

In contrast with the *a priori* hypothesis, we found no association between AD and employment status in adult life, unemployment benefits, or lower income. Other potential confounding factors, such as access to social support and social networks, might have influenced the estimates towards the null hypothesis, i.e., that there is no difference between the groups. Sex differences may also be relevant in this consideration and could not be evaluated in our study. Further, socioeconomic conditions in Swedish society have changed over the last decades, therefore a prospective study in a younger cohort is needed.

The lack of association between AD and marital status in our study in contrast with the findings of our recent cross-sectional study in a Swedish population (22) may be explained by a cohort effect; studying populations in different “cohorts” – having been born in a different time or region or having different life experiences – can alter the outcomes of studies. Period factors may also be important in this regard as prior studies have shown (31–33).

### Clinical and scientific implications

The results from this study add to the growing body of evidence which suggests that AD should be recognized and managed as a systemic condition, or at least it should be recognized that patients – especially those with severe AD – often have mental health morbidities that co-occur. This highlights the need for physicians to consider screening or monitoring for mental health comorbidities and pay attention to the impact of the disease on the social life and emotional well-being of the patient. The possibility of reducing the risk of developing mental health diseases with prompt and effective treatment of AD should be further studied.

The lack of association between AD and labour market participation in our cohort could also be a result of socioeconomic privilege. Further studies exploring this possible association between AD and labour participation are warranted. With the development of clinical registries, such as the Swedish national quality registry for AD (SwedAD), future studies can consider including validated severity measures when assessing the risk of comorbidity in patients with varying severity level.

### Conclusion

In conclusion, in this cohort study, AD was not found to be associated with a disadvantage in terms of occupation, income from work, employment, and unemployment benefits outcomes, despite increased risk of poorer mental health. Individuals with mild AD showed a lower risk of holding routine and lower technical jobs compared with men without AD, probably because of socioeconomic confounding. Individuals with AD in late adolescence seem not to differ regarding registered partnerships and marital status compared with those without AD.

## Supplementary Material

Associations of Atopic Dermatitis in Late Adolescence with Occupation, Mental Health, Income from Work, and Marital Status: A National Longitudinal Study
