# Supplementary material for: Associations of Atopic Dermatitis in Late Adolescence with Occupation, Mental Health, Income from Work, and Marital Status: A National Longitudinal Study
Source: Acta Derm Venereol. 2025 Jan 14;105:42127. doi: 10.2340/actadv.v105.42127 (PMC11748172; doi:10.2340/actadv.v105.42127)
Supplement: Associations of Atopic Dermatitis in Late Adolescence with Occupation, Mental Health, Income from Work, and Marital Status: A National Longitudinal Study [file ActaDV-105-42127-s1.pdf]

Supplementary material has been published as submitted. It has not been copyedited, or typeset by Acta Dermato-Venereologica

Appendix S1

Methods:

Data and ethics

This study used linkage of prospectively collected data in national registers from Sweden. The data from the Military conscription assessment database were linked to several registers and databases. The Longitudinal Integrated Database for Health Insurance and Labour Market Studies (Swedish acronym, LISA) compiles information on social and demographic characteristics. We extracted occupation status, unemployment, unemployment benefits, sick-leave, and marital status in 2008 from this database. The Prescribed Drug Registry (PDR), which includes data from 1 July 2005 on all pharmacy-dispensed medications, was used to obtain the information of the type of medications (Anatomical Therapeutic Chemical codes) and pharmacy dispensation dates. The Total Population Registry (TPR) was used to identify the date of birth, death and emigration. These databases are managed by Statistics Sweden (Statistikmyndigheten) and are mandatory to report to; therefore, they have complete population coverage. Data about head of household´s occupation was extracted from the 1960 Population and Housing Census. Pseudonymized unique personal identification numbers issued to all residents were used to link the data.

This study was approved by the Regional Ethics Board in Uppsala, (reference number. 2014/324 and 2019/04143). Individual consent was not required.

Covariates

Covariates assessed at time of conscription assessment were stress resilience, cognitive function, diagnoses of asthma, and any diagnosis of mental health (including depression, anxiety and schizophrenia) identified by the ICD-8 codes 493, and 290 to 315; those were considered as possible confounders and/or possible mediators on the pathway. The information of year of birth and socioeconomic index (SEI) based on occupation of the head of household when the cohort members were children was obtained from TPR and the 1960 Population and Housing Census. SEI was categorized as manual workers, agricultural workers, farm owners or managers, office workers, business owners or managers and unknown occupation. This was a priori considered as confounder, as well as the year of birth.

Statistical analysis

Cross-tabulation and median and mean were used to describe the study population and distribution of data as appropriate. The exposure was AD (any AD; mild, severe); the main outcomes were occupational group, employment status, income, unemployment benefits, and marital status in 2008. The relationship between the AD and outcomes was examined by logistic regression for dichotomous outcome (unemployment benefits), and multinomial regression for outcomes with more than two categories (occupational group, gross salary, marital status, employment status in November 2008). Coefficients, odds ratios (OR) and relative risk ratios (RRR) were estimated from these regression models, respectively, with 95% confidence intervals (95% CI). To examine the association between AD and depression and anxiety, we used Cox regression, estimating hazard ratios (HR). The proportional hazards assumption was examined using Schoenfeld residuals, and no evidence of violation was found. We stratified the analyses between AD and the outcome variables by comorbidity status to examine whether the association may differ by having any comorbid disease.

A p-value lower than 0.05 or 95% confidence intervals not including 1.00 indicated statistical significance. The analyses were conducted using Stata version 15.1.

All statistical analyses performed were adjusted for year of birth, cognitive function, any diagnosis of mental illness, and head of household´s occupation if not otherwise mentioned. Low stress resilience has been shown to be associated with increased risk of adult-onset depression, anxiety and bipolar disease in another study using the same population.[23] Therefore, stress resilience was adjusted for in some analysis to assess the extent to which it may act as an intermediate factor on the pathway, as well as AD's independent association (not through stress resilience) with depression and anxiety.

Results:

Longitudinal association between AD and mental conditions

In total, 14,868 had an outcome event (antidepressant/anxiolytic medication); the median follow-up was 162 days (IQR 48-653 days). The results from unadjusted Cox regression showed that men with AD had more often prescribed antidepressant/anxiolytic medication at the age of 50-57 years (HR 1.55 95% CI 1.32-1.81) compared with men without AD **(Table II)**. Compared with individuals without AD, the risk of prescribed antidepressants and/or anxiolytics showed a dose-response association: HR was 1.42 for men with mild AD (95% CI 1.20-1.69) and 2.46 (95% CI 1.72-3.53) for men with severe AD. These statistically significant associations remained after adjusting for year of birth, cognitive function, any diagnosis of mental illness and head of household´s occupation (HR 1.40 95% [CI 1.17-1.66] for mild AD and HR 2.41 [95% CI 1.68-3.45]

for severe AD). Further adjustment for stress resilience attenuated the estimates slightly (HR 1.37 95% CI 1.16-1.63 for mild and HR 2.36 95% CI 1.65-3.38 for severe AD respectively) but the association remained statistically significant.

When the analysis was stratified for any comorbidity in adolescence including asthma, the unadjusted HR for prescribed antidepressants/anxiolytics appeared of slightly higher magnitude for individuals with AD with comorbid diseases in adolescence (HR 1.64 95% CI 1.37-1.96) **(Table II)** . After adjustment for year of birth, cognitive function, any diagnosis of mental illness and head of household´s occupation and stress resilience the estimated hazard ratio decreased slightly but remained statistically significant (HR 1.52 95% CI 1.27-1.82).

AD and occupational socioeconomic classification

The results for the association between AD and occupational socioeconomic classification using multinomial logistic regression showed that, in 2008, men with AD had a lower risk of holding lower technical (unadjusted RRR 0.68 95% CI 0.58-0.81) and routine occupations (unadjusted RRR 0.73 95% CI 0.62-0.86) compared with men without AD in adolescence **(Table III)** . After adjusting for year of birth, cognitive function, any diagnosis of mental illness and head of household´s occupation as well as stress resilience, the association remained similar.

When we examined the association between AD and occupational socioeconomic group by AD severity, the unadjusted multinomial logistic regression model showed an inverse association between mild AD and certain employment groups; men with mild AD were less likely to have a *lower sales and service* occupation (RRR 0.79 95% CI 0.63-0.99), a *lower technical* occupation (RRR 0.66. 95%. CI 0.55-0.78) or to a *routine* occupation (RRR 0.69 95% CI 0.58-0.83) compared with men without AD **(Table IV)** . Adjustment for year of birth, cognitive function, any diagnosis of mental illness and socioeconomic index (SEI) of the head of household as well as stress resilience, slightly attenuated the associations but statistically significant associations remained for lower technical (RRR 0.71 95% CI 0.58-0.86) and routine occupations (RRR 0.71 95% CI 0.59-0.87).

When the analysis was stratified by comorbidity, the unadjusted relative risk ratio for holding a lower technical job was 0.60 (95% CI 0.45-0.81) and for routine job was 0.58 (95% CI 0.42-0.78) - with large employers/higher managers as the reference category- for men with AD without comorbidities **(Table SI)**. Adjustment for year of birth, stress resilience, cognitive function, any diagnosis of mental illness and head of household´s occupation attenuated the magnitude of the associations between AD without comorbidity and routine occupations which remained borderline statistically significant (RRR 0.70 95% CI 0.50-0.98) whereas the association between AD without comorbidity and lower technical occupations

became no longer statistically significant (RRR 0.72 95% 0.52-1.00). Men with AD with at least one comorbidity were found to have a lowerrelative risk ratio of holding lower technical (RRR 0.73 95% CI 0.59-0.89) or routine occupation (RRR 0.81 95% CI 0.66-0.98) in both crude and adjusted analyses (RRR for lower technical jobs 0.73 95% CI 0.59-0.91 and RRR for routine jobs 0.76 95% CI 0.61-0.95).

AD, labour market participation and unemployment benefits

No association between AD and labour market participation in November 2008 was found in unadjusted (RRR 0.88 95% CI 0.67-1.15) or adjusted analyses (aRRR 0.87 95% CI 0.67-1.14). (**Table III)** Analysis by AD severity showed an unadjusted RRR for not worked during the year 0.79 for men with mild AD (95% CI 0.58-1.07) and 1.55 for individuals with severe disease (95% CI 0.86-2.78). Adjusted analyses for year of birth, cognitive function, any diagnose of mental illness and head of household´s occupation as well as further adjustment including stress resilience did not alter these estimates notably which remained not statistically significant as shown in **Table SIV**. Stratification for comorbidity did not alter the estimates either. The unadjusted RRR of not working in 2008 for men with AD without comorbidity was 0.71 (95% CI 0.41-1.24) whereas for men with AD and comorbidity was higher but not statistically significant (RRR 0.95 95% CI (0.70-1.29) (**Tables SI and SII).**

AD and unemployment benefits

Table III shows that AD was not statistically significantly associated with higher risk of receiving unemployment benefits in crude analysis, (OR 0.93 95% CI 0.71-1.21). Adjustment for year of birth, cognitive function, any diagnose of mental illness and SEI of the head of household as well as additional adjustment including stress resilience did not alter the estimate notably (RRR 0.95 95% CI 0.73-1.24 and RRR 0.93 95% CI 0.71-1.21, respectively). Analyses by disease severity showed an unadjusted odds ratio of 0.95 of receiving unemployment benefits for men with mild AD (95 % CI 0.72-1.25) and of 0.77 for men with severe disease (95% CI 0.34-1.73) compared with persons without AD. See **Table III** for more details. Analyses by comorbidity status did not show any statistically significant association between AD with or without comorbidities and unemployment benefits; **Tables SI–SIII.**

AD and gross salary

Compared with a lower income, the unadjusted multinomial regression model showed no statistically significant increased relative risk of an income of 420 000 SEK or higher for persons with AD in adolescence (RRR 1.13, 95% CI 0.98-1.30) compared with individuals without AD **(Table III)** . Adjustment for year of

birth, cognitive function, any diagnose of mental illness and SEI of the head of household as well as additional adjustment including stress resilience did not make noticeable changes in the estimates. Unadjusted analysis by disease severity found that persons with mild AD had a higher relative risk ratio (1.17 95% CI 1.00-1.36) of a high income (>420,000 SEK) compared to persons without AD while the relative risk ratio of a high income for persons with severe AD was 0.86 (95% CI 0.57-1.31). After adjustment, AD was not associated with gross salary, in the analysis of whole population as well as that stratifying by comorbidity (**Tables SII and SIII)** .

AD and marital status

The unadjusted analysis found that AD was not statistically significantly associated with marital status as shown in **Table III**. The unadjusted multinomial logistic regression produced a relative risk ratio of 1.07 (95% CI 0.95-1.20) for never been married and 0.94 (95% CI 0.81-1.09) for been divorced respectively for men with AD -with married as reference category- compared with those without AD. After adjustment for year of birth, cognitive function, any diagnosis of mental illness and head of household´s occupation, the estimates did not alter notably and remained statistically not significant. There was no statistically significant association between the severity of AD and marital status; for divorced unadjusted RRR 1.05 for severe AD (95% CI 0.71-1.57) and RRR 0.93 for mild AD (95% CI 0.79-1.08), **Table IV**. Crude and adjusted analyses stratified by disease severity and comorbidities did not alter the estimates notable which remained statistically not significant. The results are shown in **Tables SII and SIII**.

Table SI. Association between AD with and without any comorbidity and occupation, gross salary, unemployment benefits, employment status and marital status

Outcome variable	Category	AD without comorbidity		AD with comorbidity	
		**Unadjusted RRR (95 % CI)**	**Adjusted RRR (95 % CI) ***	**Unadjusted RRR (95 % CI)**	**Adjusted RRR (95 % CI) ***
**Occupational socioeconomic classification**					
	Large employers, higher managers/professionals	Reference	Reference	Reference	Reference
	Lower managers/professionals, higher supervisory/technicians	0.72 (0.55 – 0.96)	0.76 (0.57 – 1.01)	0.94 (0.78 – 1.13)	0.94 (0.78 – 1.14)
	Intermediate occupations	0.71 (0.50 – 1.03)	0.75 (0.52 – 1.09)	0.85 (0.67 – 1.09)	0.86 (0.67 – 1.10)
	Lower supervisors and technicians	0.49 (0.15 – 1.53)	0.51 (0.16 – 1.62)	0.98 (0.54 – 1.75)	0.95 (0.53 – 1.71)
	Lower sales and service	0.53 (0.34 – 0.82)	0.60 (0.38 – 0.94)	1.04 (0.81 – 1.32)	0.98 (0.76 – 1.27)
	Lower technical	0.60 (0.45 – 0.81)	0.72 (0.52 – 1.00)	0.73 (0.59 – 0.89)	0.73 (0.59 – 0.91)
	Routine	0.58 (0.42 – 0.78)	0.70 (0.50 – 0.98)	0.81 (0.66 – 0.98)	0.76 (0.61 – 0.95)
**Gross salary(quartiles)**					
	≤ 244,500	Reference	Reference	Reference	Reference
	244,600-322,300	1.04 (0.78 – 1.39)	1.00 (0.74 – 1.33)	0.93 (0.78 – 1.10)	0.96 (0.80 – 1.14)
	322,400-420,000	1.24 (0.94 – 1.63)	1.09 (0.82 – 1.44)	0.93 (0.78 – 1.10)	0.97 (0.81 – 1.16)
	> 420,000	1.28 (0.97 – 1.69)	1.01 (0.76 – 1.35)	1.07 (0.91 – 1.27)	1.13 (0.94 – 1.3)
**Unemployment benefits** **					
	No benefits	Reference	Reference	Reference	Reference
	Benefits	0.84 (0.50 – 1.41)	0.93 (0.55 – 1.55)	0.96 (0.71 – 1.31)	0.93 (0.68 – 1.26)
**Employment status**					
	Working	Reference	Reference	Reference	Reference
	Not working in November, has worked during the year	0.88 (0.47 – 1.64)	1.03 (0.55 – 1.94)	0.81 (0.53 – 1.22)	0.80 (0.53 – 1.22)
	Not working in November, has not worked during the year	0.71 (0.41 – 1.24)	0.84 (0.48 – 1.46)	0.95 (0.70 – 1.29)	0.88 (0.65 – 1.20)
**Marital status**					
	Married	Reference	Reference	Reference	Reference
	Never married	0.94 (0.75 – 1.19)	1.00 (0.79 – 1.26)	1.12 (0.97 – 1.29)	1.03 (0.89 – 1.19)
	Divorced	0.80 (0.60 – 1.06)	0.83 (0.62 – 1.11)	1.00 (0.84 – 1.19)	0.97 (0.82 – 1.15)

**Adjusted for year of birth, stress resilience, cognitive function, any diagnose of mental illness and head of household's occupation.*
***Logistic regression (OR, 95 % CI).*
RRR Relative Risk Ratio; CI Confidence Interval

Table SII. Association between AD without any comorbidity, occupation, salary, unemployment benefits and marital status

Outcome variable	Category	Mild AD Unadjusted RRR (95 % CI)	Severe AD Unadjusted RRR (95 % CI)	Mild AD Adjusted RRR (95 % CI) *	Severe AD Adjusted RRR (95 % CI) *
**Occupational socioeconomic classification**					
	Large employers, higher managers/professionals	Reference	Reference	Reference	Reference
	Lower managers/professionals, higher supervisory/technicians	0.72 (0.54 – 0.97)	0.71 (0.28 – 1.80)	0.77 (0.57 – 1.04)	0.71 (0.28 – 1.79)
	Intermediate occupations	0.60 (0.40 – 0.90)	1.88 (0.76 – 4.62)	0.63 (0.42 – 0.95)	1.84 (0.74 – 4.61)
	Lower supervisors and technicians	0.54 (0.17 – 1.69)	-	0.57 (0.18 – 1.80)	-
	Lower sales and service	0.52 (0.33 – 0.82)	0.66 (0.18 – 2.44)	0.59 (0.37 – 0.95)	0.67 (0.18 – 2.58)
	Lower technical	0.60 (0.44 – 0.81)	0.65 (0.25 – 1.68)	0.73 (0.52 – 1.03)	0.68 (0.25 – 1.89)
	Routine	0.54 (0.38 – 0.74)	0.98 (0.40 – 2.35)	0.66 (0.46 – 0.95)	1.06 (0.41 – 2.77)
**Gross salary (quartiles)**					
	≤ 244,500	Reference	Reference	Reference	Reference
	244,600-322,300	1.02 (0.75 – 1.40)	1.15 (0.53 – 2.49)	0.98 (0.72 – 1.34)	1.09 (0.50 – 2.36)
	322,400-420,000	1.28 (0.95 – 1.71)	0.99 (0.44 – 2.19)	1.12 (0.83 – 1.51)	0.87 (0.39 – 1.95)
	> 420,000	1.33 (0.99 – 1.78)	0.99 (0.44 – 2.20)	1.04 (0.76 – 1.41)	0.84 (0.37 – 1.94)
**Unemployment benefits ****					
	No benefits	Reference	Reference	Reference	Reference
	Benefits	0.83 (0.47 – 1.44)	0.93 (0.23 – 3.83)	0.92 (0.53 – 1.60)	1.00 (0.24 – 4.12)
**Employment status in November**					
	Working	Reference	Reference	Reference	Reference
	Not working in November, has worked during the year	0.90 (0.46 – 1.74)	0.73 (0.10 – 5.30)	1.06 (0.55 – 2.06)	0.85 (0.12 – 6.17)
	Not working in November, has not worked during the year	0.69 (0.38 – 1.25)	0.92 (0.22 – 3.77)	0.81 (0.44 – 1.48)	1.03 (0.25 – 4.28)
**Marital status**					
	Married	Reference	Reference	Reference	Reference
	Never married	0.95 (0.74 – 1.22)	0.89 (0.47 – 1.69)	1.02 (0.79 – 1.31)	0.90 (0.47 – 1.72)
	Divorced	0.88 (0.65 – 1.18)	0.31 (0.09 – 1.01)	0.91 (0.68 – 1.23)	0.31 (0.10 – 1.02)

** Adjusted for year of birth, stress resilience, cognitive function, any diagnose of mental illness and head of household's occupation.*
***Logistic regression (OR, 95 % CI).*
RRR Relative Risk Ratio; CI Confidence Interval

Table SIII. Association between AD with any comorbidity, and occupation, salary, unemployment benefits and marital status

Outcome variable	Category	Mild AD Unadjusted RRR (95 % CI)	Severe AD Unadjusted RRR (95 % CI)	Mild AD Adjusted RRR (95 % CI) *	Severe AD Adjusted RRR (95 % CI) *
**Occupational socioeconomic classification**					
	Large employers, higher managers/professionals	Reference	Reference	Reference	Reference
	Lower managers/professionals, higher supervisory/technicians	0.89 (0.73 – 1.09)	1.35 (0.76 – 2.39)	0.91 (0.74 – 1.11)	1.30 (0.73 – 2.32)
	Intermediate occupations	0.88 (0.68 – 1.13)	0.56 (0.22 – 1.42)	0.89 (0.69 – 1.16)	0.54 (0.21 – 1.37)
	Lower supervisors and technicians	0.99 (0.54 – 1.82)	0.87 (0.12 – 6.54)	0.97 (0.52 – 1.78)	0.81 (0.11 – 6.07)
	Lower sales and service	0.93 (0.71 – 1.21)	2.09 (1.10 – 3.98)	0.89 (0.68 – 1.18)	1.80 (0.91 – 3.56)
	Lower technical	0.69 (0.56 – 0.85)	1.09 (0.60 – 1.99)	0.70 (0.56 – 0.88)	1.02 (0.53 – 1.94)
	Routine	0.77 (0.62 – 0.95)	1.15 (0.63 – 2.10)	0.74 (0.59 – 0.94)	0.98 (0.51 – 1.89)
**Gross salary (quartiles)**					
	≤ 244,500	Reference	Reference	Reference	Reference
	244,600-322,300	0.88 (0.73 – 1.07)	1.21 (0.78 – 1.89)	0.91 (0.75 – 1.10)	1.23 (0.79 – 1.93)
	322,400-420,000	0.96 (0.80 – 1.16)	0.68 (0.40 – 1.14)	1.01 (0.84 – 1.22)	0.71 (0.42 – 1.21)
	> 420,000	1.11 (0.93 – 1.33)	0.82 (0.50 – 1.34)	1.16 (0.96 – 1.40)	0.92 (0.55 – 1.56)
**Unemployment benefits ***					
	No benefits	Reference	Reference	Reference	Reference
	Benefits	1.00 (0.72 – 1.38)	0.70 (0.26 – 1.91)	0.98 (0.71 – 1.35)	0.64 (0.23 – 1.73)
**Employment status**					
	Working	Reference	Reference	Reference	Reference
	Not working in November, has worked during the year	0.88 (0.58 – 1.35)	0.29 (0.04 – 2.05)	0.89 (0.58 – 1.36)	0.25 (0.04 – 1.81)
	Not working in November, has not worked during the year	0.83 (0.59 – 1.18)	1.80 (0.94 – 3.42)	0.78 (0.55 – 1.11)	1.55 (0.80 – 2.97)
**Marital status**					
	Married	Reference	Reference	Reference	Reference
	Never married	1.12 (0.96 – 1.30)	1.15 (0.76 – 1.73)	1.03 (0.88 – 1.20)	1.05 (0.69 – 1.59)
	Divorced	0.95 (0.79 – 1.14)	1.42 (0.92 – 2.20)	0.92 (0.76 – 1.11)	1.34 (0.86 – 2.08)

** Adjusted for year of birth, stress resilience, cognitive function, any diagnose of mental illness and head of household's occupation.*
***Logistic regression (OR, 95 % CI).*
RRR Relative Risk Ratio; CI Confidence Interval
